# Ectopic Thoracic Kidney and End-Stage Renal Disease in a 38-Year-Old Nigerian

**DOI:** 10.1155/2013/158494

**Published:** 2013-05-02

**Authors:** U. E. Ekrikpo, E. E. Effa, E. E. Akpan

**Affiliations:** ^1^Department of Internal Medicine, University of Uyo Teaching Hospital, PMB 1136, Uyo, Nigeria; ^2^Department of Internal Medicine, University of Calabar Teaching Hospital, PMB 1278, Calabar, Nigeria

## Abstract

This patient is a 38-year-old housewife who presented with a one-month history of difficulty, in breathing, chest pain and bilateral leg swelling and had a blood pressure of 260/150 mmHg, features of malignant hypertension and hypertensive heart disease. Chest CT scan revealed a chest location of the left kidney. She also had elevated serum urea and creatinine and proteinuria (++). The right kidney was normally located with loss of corticomedullary differentiation. She is on maintenance haemodialysis and is being worked up for possible left nephrectomy.

## 1. Introduction

Renal ectopias are relatively common congenital anomalies believed to occur in about 1/1000 live births, though only about 10% are usually detected [1]. Ectopic thoracic kidneys, on the other hand, are very rare anomalies and constitute less than 5% of renal ectopias [2–4]. In Nigeria, only one case has been documented [5]. Many of the cases of intrathoracic kidneys reported have been asymptomatic and only discovered during routine medical checks or having mild respiratory symptoms.

Here we report a case of a 38-year-old Nigerian female with a left intrathoracic ectopic kidney and markedly elevated blood pressure leading to renal failure.

## 2. Case Presentation

This patient is a 38-year-old housewife who was referred from the general out-patient clinic on account of a one-month history of recurrent difficulty in breathing, associated with chest pain, recurrent headaches, blurring of vision, and dizziness with blood pressure of 260/150 mmHg. There was a history of orthopnoea and paroxysmal nocturnal dyspnoea. She had no associated cough but had a mild centrally located chest pain. There was a history of bilateral leg swelling which has been progressively worsening and associated with diminution in urine volume. She was diagnosed as hypertensive five years before presentation but had not been compliant with antihypertensive medications. There was no known family history of hypertension.

Examination showed a young woman in respiratory distress, pale, afebrile (temperature 36.8°C), bilateral pitting leg oedema up to the knee with no sacral oedema. Waist circumference was 78 cm, hip circumference 80 cm, and a body mass index (BMI) 23.8 kg/m^2^. 

Her respiratory rate was 50 cycles/minute with reduced breath sounds in the left lower lung zones and bibasal fine crepitations. The pulse rate was 118 beats/minute, normal volume and regular. There was clinical evidence of arterial wall thickening. Blood pressure was 220/120 mmHg with a fourth heart sound and loud aortic component of the second heart sound. She also had grade 3 hypertensive retinopathy on fundoscopy and asterixis but no lateralizing signs.

### 2.1. Clinical Diagnosis

An initial diagnosis of acute-on-chronic renal failure secondary to hypertensive nephrosclerosis, precipitated by malignant hypertension, was made.

Results of investigations are shown as follows.

**Figure 1:**
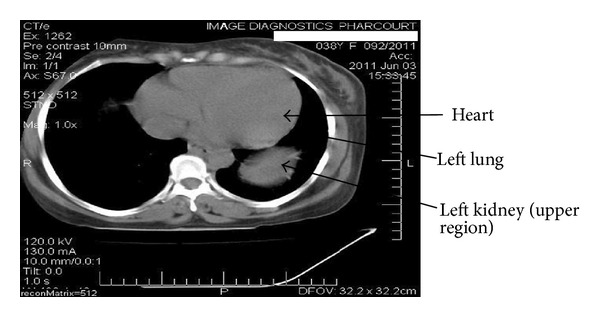
The left ectopic kidney found at T5/T6 transverse level.

**Figure 2:**
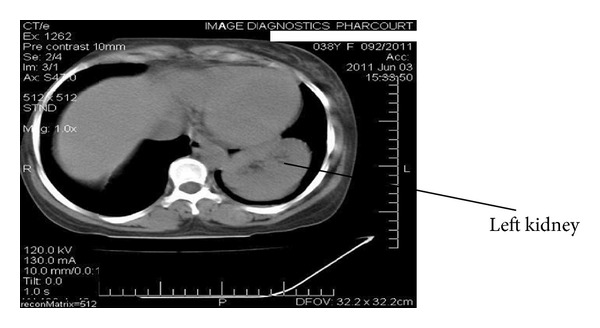
The left kidney in the thoracic cavity at T9 thoracic level. Notice the right kidney is yet to be visualized.

**Figure 3:**
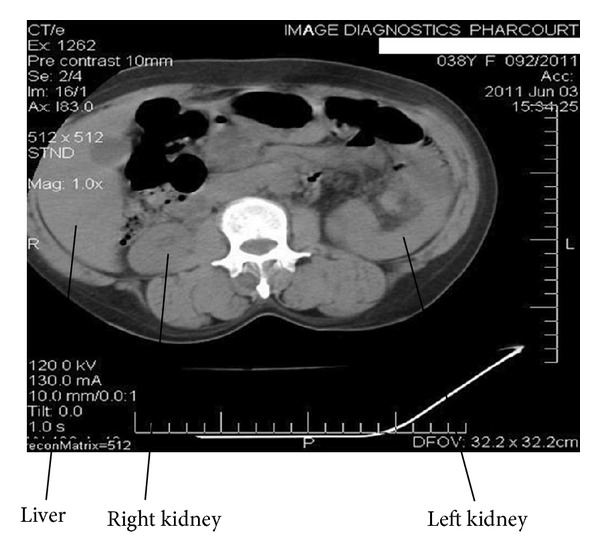
This CT slide shows both kidneys and the liver.

- Results of investigations done: E/U/C (predialysis).
  - Creatinine: 900 *μ*mol/L (53–115).
  - Urea: 30 mmol/L (2.1–7.1).
  - Na: 136 mmol/L (135–145).
  - K: 4.0 mmol/L (3.2–5.0).
  - Cl: 98 mmol/L (96–108).
  - HCO_3_: 28 mmol/L (22–28).
- HBsAg, anti-HCV, and HIV: negative.
- Urinalysis showed ++ proteinuria, no haematuria.
- Renal ultrasound scan and renal artery Doppler: renal USS/Doppler-right kidney measured 8.63 × 4.59 × 5.07 cm. There was loss of corticomedullary differentiation. Doppler insonation of the renal artery showed a velocity of 73.9 cm/second and a resistive index of 0.9 suggesting renal intraparenchymal disease. The upper margin of the left kidney appeared to be intrathoracic and could not be well deciphered. The echotexture was hypoechoic with complete loss of corticomedullary differentiation. Doppler insonation of the left renal artery showed a velocity of 195 cm/min consistent with a stenotic artery.
- On the basis of the finding suggestive of left renal artery stenosis, the plan was to do a renal artery angiogram, but the risk of contrast-induced nephropathy precluded this investigation.
- Abdominal CT scan report: the scanogram showed a left intrathoracic well-marginated upper border mass obscuring the left hemidiaphragm medially and obscuring the cardiac apex. Contiguous slices showed the left kidney to be in an abnormal location in the left hemithorax sitting between the cardiac silhouette and spleen. The right kidney was in its normal location with major blood vessels appearing normal. The left kidney was slightly bigger than the right one, (see Figures 1, 2, and 3).
- Captopril renogram.
  - Right kidney: intact perfusion followed by almost uniform uptake of radiotracer.
  - Left kidney: intact perfusion followed by nonuniform uptake of radiotracer. Its upper moiety is truncated out of field of view of the camera, therefore, making computation of differential renal function impossible.
  - High background activity indicating reduced renal function in the patient. This background activity increased after administration of captopril.
  - Tc-99 m DMSA radionuclide scan was suggested but patient declined.

A possible diagnosis of left intrathoracic ectopic kidney with chronic kidney disease secondary to possible renovascular hypertension was made.

She is now on maintenance haemodialysis, with resolution of uraemic symptoms. Leg swelling, orthopnoea, and paroxysmal nocturnal dyspnoea had completely resolved; her blood pressure has been between 120/88 and 150/104 mmHg with serum creatinine of 248–380 *μ*mol/L. Her medications included alpha methyldopa at 1 g bd; Amlodipine 10 mg daily; Brinerdin 1 tab bd; Atenolol 40 mg daily; and oral furosemide 80 mg twice daily.

## 3. Discussion

An adult with a thoracic left renal ectopia, features of renovascular hypertension and chronic kidney disease secondary to hypertension, has been presented.

Many theories have been propounded for the possible aetiology of ectopic thoracic kidneys [3, 6]. A possible aetiology is a delayed closure of the diaphragmatic anlage allowing protracted ascent of the kidneys beyond its normal position. Another possibility is a delay in the caudal migration of the mesonephron leading to a diaphragmatic defect which allows the kidneys to be displaced cranially. It could also be an ab initio high cephalic establishment of the renal germ or delay in the joining of the ureteric bud to the metanephron leading to late differentiation of metanephric tissue which in turn leads to accentuation of the ascending process.

The youngest age recorded for the identification of an ectopic thoracic kidney was in a 13-day-old premature infant in Israel [7]; it has also been discovered in the elderly with a report of a 75-year-old woman, and the average age of reported cases was 41 years [8]. Our patient was 38 years old at the time of diagnosis. The earliest clinical case report was by Wolfromm in 1940, but, in 1927, Gruber had reported thoracic kidneys in fetuses and stillborn babies [8]. Thoracic ectopic kidneys occur with a slight male preponderance (1.5 : 1) with the left usually more affected [3, 9]. This patient had a left thoracic ectopic kidney. Bilateral thoracic ectopic kidneys have also been reported [4]. They are usually asymptomatic and only discovered on routine medical examinations, though there is a report of chest pain occurring in a patient with a left intrathoracic kidney [9]. In this patient, investigations for a possible renovascular cause for her very high blood pressure recordings led to the discovery of a left thoracic ectopic kidney.

Renal vascular abnormalities abound in patients with renal ectopia [10]. This may range from very rare conditions, like entrapment of the renal artery by the diaphragmatic crura to possession of multiple aberrant arteries, to the more common stenosis of the renal vessels due to plaque deposition. Doppler ultrasound of the renal vessels had shown significant reduction in flow of the left renal arteries, suggesting that the hypertension she had may have been of renovascular origin. If this was the case, she may have benefitted from a left nephrectomy if the condition was discovered before she had developed established chronic renal failure. 

The chronic kidney disease in this patient appears to be due to renovascular hypertension. The patient presented with resistant hypertension requiring 5 antihypertensives and episodes of recurrent pulmonary oedema; developed hypertension at a relatively early age; and had grade III hypertensive retinopathy. In classical renovascular hypertension, there is significant reduction in the size of the affected kidney (ischaemic nephropathy) compared to the contralateral kidney. This was not the case here as the offending kidney appeared to be the one with an ectopic location and had a relatively larger size albeit with complete loss of corticomedullary differentiation. Increased plasma renin activity can be helpful in the diagnosis of renovascular hypertension especially if blood from the ipsilateral renal vein is sampled for the assay, but this might not have been of much help here as the patient had already developed advanced chronic kidney disease and would have elevated plasma renin activity. One would now resort to diagnostic imaging to help confirming the diagnosis. 

The results from the renal ultrasound scan and that of the CT scan appeared to be contradictory. It appears the ultrasonographer erroneously gave the dimensions of the left kidney to be much smaller than it actually was—a case of operator-dependent ultrasound diagnosis error. Despite the error in assessing the size of the ectopic kidney, the Doppler scan showed a significantly reduced left renal artery velocity—the so-called pulsus parvus [11]—which may be diagnostic of renal artery stenosis. Also, the resistive index (RI) of 0.9 indicates that the patient may not benefit from a revascularization procedure as RI of ≥0.8 has been found to indicate poor prognosis in reducing hypertension after revascularization [11]. The captopril renogram was inconclusive and this patient would have benefitted from a magnetic resonance angiography which would have aided in measuring the GFR and absolute blood flow in the affected vessel, but she had run out of funds. Spiral CT angiography was contraindicated because of the risk of contrast nephropathy in this patient who had already developed elevated serum creatinine.

In conclusion, this report highlights the presence of this rare congenital renal anomaly in our population and underlines the importance of early imaging studies of the kidneys in every young patient with chronic kidney disease because of the possibility of identifying treatable causes of hypertension.
